# The SPX protein family in plants: from phosphate sensors to multifunctional signaling hubs

**DOI:** 10.1007/s44154-026-00307-3

**Published:** 2026-05-08

**Authors:** Shenghong Ge, Kai Yuan, Mingguang Lei

**Affiliations:** 1 School of Life Sciences, Central South University, Changsha, Hunan China; 2 College of Life and Environmental Sciences, Hangzhou Normal University, Hangzhou, 311121 China

**Keywords:** SPX domain, PHR transcription factors, Phosphate signaling, Inositol pyrophosphates, Biotic and abiotic stresses

## Abstract

Phosphorus (P) is an essential macronutrient for plant growth and development, yet its limited availability in soil severely constrains crop productivity. To cope with phosphate (Pi) deficiency, plants have evolved a sophisticated signaling network centered on SPX domain proteins, which serve as central regulators of Pi homeostasis. Recent breakthrough structural studies have revolutionized our understanding of these proteins, revealing their function as cellular Pi sensors through binding of the inositol pyrophosphate InsP_8_. This review synthesizes current knowledge of SPX protein molecular structures, evolution, and functions within the Pi signaling network. We detail their sensing mechanism, focusing on inositol pyrophosphate binding and the subsequent control of PHR activity and phosphate starvation response (PSR) gene expression. Recent cryo-electron microscopy structures of rice SPX1-PHR2, *Arabidopsis* PHO1;H1, and human XPR1 have provided unprecedented insights into phosphate transport mechanisms and SPX domain regulation. We also discuss emerging functions of SPX proteins in coordinating arbuscular mycorrhizal symbiosis, plant immunity, nitrogen-phosphorus balance, and cold stress responses, highlighting their broad significance in plant biology. Finally, we discuss key challenges and future research directions crucial for translating these mechanistic insights into innovative strategies to enhance phosphorus use efficiency (PUE) in crops, including structure-guided protein engineering approaches.

**Supplementary Information:**

The online version contains supplementary material available at 10.1007/s44154-026-00307-3.

## Introduction

Phosphorus is a fundamental element of life, serving as a structural component of essential biomolecules including nucleic acids, phospholipids, and proteins. It is also indispensable for numerous cellular processes, including energy metabolism, photosynthesis, and signal transduction (Vance et al. 2003; Bowler et al. 2010). Plants primarily acquire phosphorus from soil as inorganic phosphate (Pi). However, soil phosphorus is highly prone to fixation, forming insoluble complexes or organic compounds that are not readily accessible to plants. Consequently, phosphorus availability represents a major constraint on crop productivity in most agricultural systems worldwide (Chiou and Lin 2011; Lynch 2011).

In response to Pi deficiency, plants have evolved sophisticated adaptive mechanisms that collectively enhance the acquisition and utilization of limited Pi resources. These adaptations primarily include: (1) remodeling of root system architecture (RSA) through enhanced root hair and lateral root development to maximize soil exploration and absorptive surface area (Lopez-Bucio et al. 2003; Peret et al. 2014); (2) secretion of root exudates, including organic acids and acid phosphatases, into the rhizosphere to mobilize fixed Pi and mineralize organic phosphorus (Tran et al. 2010); (3) induction of internal protective responses, such as anthocyanin accumulation, to mitigate oxidative stress and maintain cellular integrity (Wang et al. 2011); and (4) coordinated regulation of PSR genes to reprogram Pi uptake, redistribution, and cellular Pi homeostasis (Puga et al. 2017; Wu et al. 2013).

Within this finely tuned Pi signaling network, SPX domain proteins have emerged as central regulators of Pi homeostasis. The SPX domain represents an evolutionarily conserved hydrophilic module that is critical for maintaining Pi homeostasis across diverse eukaryotic lineages. Recent structural studies have revealed that SPX domains function as molecular sensors that directly bind inositol pyrophosphates (PP-InsPs), particularly InsP_8_, which serve as intracellular messengers reflecting cellular phosphate status (Dong et al. 2019; Zhu et al. 2019). In model plants such as *Arabidopsis* and rice, SPX proteins sense cellular Pi levels through their SPX domain and directly interact with the central transcription factor PHR1 in a Pi-dependent manner. This interaction inhibits PHR1 activity to control the expression of PSR genes, creating a negative feedback loop that maintains phosphate homeostasis (Puga et al. 2014; Wang et al. 2014). Beyond this conserved core mechanism, the regulatory modes of SPX proteins exhibit considerable diversity across species. For instance, SlSPX5 in tomato interacts with the transcription factor SlPHL1 independently of Pi levels, suppressing the PSR by sequestering it in the cytoplasm (Ge et al. 2025). SPX proteins are classified into four major subfamilies based on their distinct C-terminal domains: SPX, SPX-EXS, SPX-MFS, and SPX-RING. Together, these SPX proteins constitute a multi-layered and functionally integrated regulatory network that orchestrates phosphorus nutrition (Liu et al. 2018).

Plant growth and development are governed by complex, interconnected signaling networks, where phosphorus signaling does not operate in isolation. Rather, it interacts with various environmental and developmental cues, including other nutrients (e.g., carbon, nitrogen), abiotic stresses (e.g., drought, salinity, cold), and biotic interactions (e.g., pathogen attack, mycorrhizal symbiosis). Emerging evidence shows that SPX domain proteins serve as critical integration hubs within this network. For instance, they directly contribute to the coordinated regulation of nitrogen-phosphorus balance (Ueda et al. 2020; Hu et al. 2019), modulation of responses to low temperature and salt stress (Zhao et al. 2009; Du et al. 2024), and establishment of arbuscular mycorrhizal symbiosis (Shi et al. 2021). These findings position SPX proteins as central integrators that merge internal nutrient status with diverse environmental cues, providing new perspectives for understanding plant environmental adaptation and opening novel research avenues.

This review aims to synthesize current understanding of the structural and evolutionary features of the plant SPX gene family, with particular emphasis on how functional diversification has occurred across subfamilies. We provide a detailed analysis of the molecular mechanisms underlying their role as phosphate sensors, including their specific recognition of inositol pyrophosphate signals and the dynamics of their Pi-dependent conformational changes. Building on insights from comparative genomics and functional studies, we consolidate their central role within the Pi signaling network while expanding the discussion to encompass the integrative functions of SPX proteins in coupling phosphate signaling with responses to biotic and abiotic stresses, as well as beneficial symbiotic interactions. Synthesizing the latest evidence from structural biology, systems biology, and applied research, we propose a unified "SPX-Centered Regulatory Network" model that captures the multifaceted roles of these proteins in plant adaptation. Ultimately, this review aims to identify key genetic targets and molecular mechanisms for enhancing phosphorus use efficiency (PUE) in crops, thereby contributing to the theoretical foundation required for advancing sustainable agricultural practices in phosphorus-limited environments.

## Molecular features and evolution of the SPX domain protein family

### Discovery and nomenclature of SPX domain Proteins

The SPX domain derives its name from three founding members: the Suppressor of Yeast *gpa1* (SYG1), yeast Phosphatase 81 (PHO81), and human xenotropic and polytropic retrovirus receptor 1 (XPR1) (Secco et al. 2012; Hürlimann et al. 2009; Wang et al. 2004). Functional characterization of these proteins revealed the SPX domain's involvement in diverse fundamental biological processes. In yeast, SYG1 modulates G-protein signaling, while PHO81 acts as a cyclin-dependent kinase inhibitor directly involved in phosphate sensing. In mammals, XPR1 serves dual roles as both a retroviral receptor and a phosphate transporter (Spain et al. 1995; Yan et al. 2024). Bioinformatic analyses identified a conserved hydrophilic region of ~ 165 amino acids at the N-terminus of these proteins, which defines the signature SPX domain. Subsequent investigations across plants and diverse eukaryotes have firmly established the SPX domain as an conserved module for cellular phosphate homeostasis, underscoring its remarkable functional conservation throughout evolution (Collins et al. 2024; Jung et al. 2018; Secco et al. 2012; Yan et al. 2024).

### Structural characteristics of SPX proteins

The phosphate-sensing function of the SPX domain is directly determined by its distinctive structural architecture. The domain typically consists of three sub-domains of 30–40 amino acids each, connected by flexible linker regions (Liu et al. 2018). This arrangement generates a unique three-dimensional fold characterized by a highly basic, positively charged surface at its core, formed primarily by a three-helix bundle together with an N-terminal α-hairpin motif (Wild et al. 2016). In plants, SPX domain proteins are classified into four subfamilies based on their C-terminal domain architecture: SPX-only proteins (containing solely the SPX domain), SPX-EXS proteins (containing both SPX and EXS domains), SPX-MFS proteins (featuring SPX and a Major Facilitator Superfamily domain), and SPX-RING proteins (possessing SPX along with a RING-type zinc finger domain). This structural diversity dictates their distinct biochemical functions and mechanistic roles. The first subfamily, exemplified by AtSPX1/2 in *Arabidopsis* and their orthologs in rice, functions as phosphate sensors that perceive cellular phosphate status via their conserved SPX domain. They directly interact with and inhibit the transcriptional activity of AtPHR1/OsPHR2 in a Pi-dependent manner, thereby transducing the phosphate signal (Wang et al. 2014; Puga et al. 2014; Lv et al. 2014). This sensing mechanism is mediated by inositol pyrophosphate InsP_8_, a conserved regulator of phosphate homeostasis in both plants and animals. InsP_8_ binds directly to the SPX domain, inducing a conformational change that enhances the SPX-PHR interaction. Thus, this mechanism precisely couples intracellular phosphate status to transcriptional control (Wang et al. 2025; Dong et al. 2019; Li et al. 2020; Zhou et al. 2021).

The other SPX subfamilies also play crucial roles in maintaining Pi homeostasis. SPX-EXS (Class II) proteins, such as PHOSPHATE1 (PHO1), mediate phosphate loading and long-distance transport (Wang et al. 2004; Fang et al. 2025). The SPX-MFS (Class III) family (also known as PHT5/VPT) regulates phosphate partitioning between the vacuole and cytosol to maintain cytosolic Pi homeostasis (Liu et al. 2016; Wang et al. 2015). SPX-RING (Class IV) proteins, such as NLA (NITROGEN LIMITATION ADAPTATION), function as E3 ubiquitin ligases that regulate the stability of plasma membrane-localized phosphate transporters (Park et al. 2014; Yang et al. 2017). Together, these four subfamilies constitute a functionally integrated network that coordinates Pi sensing, signaling, transport, and storage across different cellular compartments and tissues.

### Phylogenetic analysis of the *SPX* gene family

**Fig. 1:**
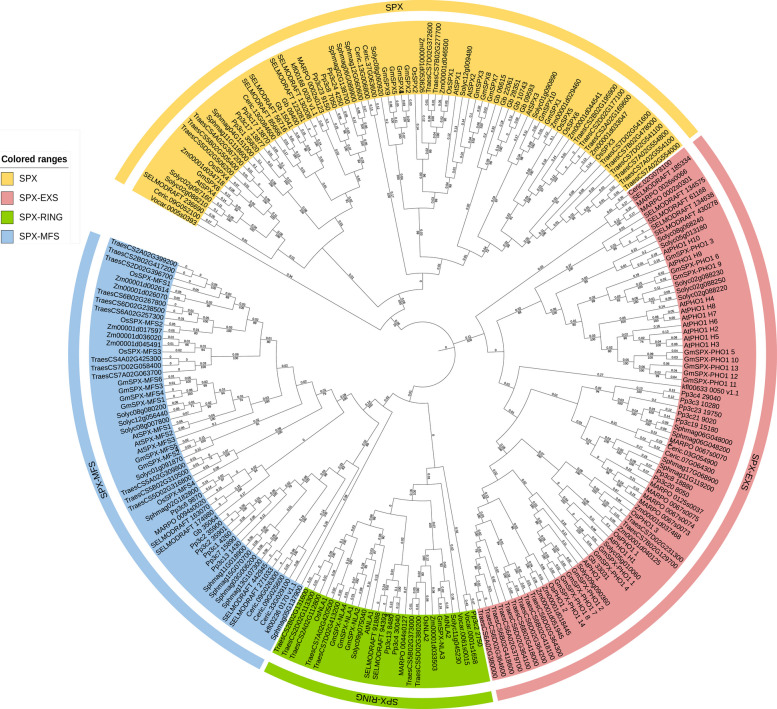
Phylogenetic analysis of SPX domain proteins across plant lineages. Phylogenetic tree showing the evolutionary relationships of SPX domain proteins from representative species spanning major plant lineages, including chlorophytes, streptophyte algae, bryophytes, ferns, gymnosperms, and angiosperms. SPX domain-containing protein sequences were identified by BLAST searches against public databases (Phytozome, NCBI, and Ensembl Plants) using conserved SPX domain sequences as queries, followed by manual curation to confirm domain integrity. The four major subfamilies (SPX, SPX-EXS, SPX-MFS, and SPX-RING) are indicated by different colors. Bootstrap values are shown at branch nodes. The phylogenetic tree was performed in MEGA11 using the neighbor-joining method with 1000 bootstrap replicates. The tree was rooted and visualized using iTOL (https://itol.embl.de/login.cgi) (Letunic and Bork 2021)

To elucidate the evolutionary trajectory of the *SPX* gene family, we performed a comprehensive phylogenetic analysis using SPX domain-containing sequences from a broad range of green plants (Supplementary Dataset1). Our analysis encompassed representative species spanning major plant lineages: angiosperm eudicots (*Arabidopsis thaliana*, *Solanum lycopersicum* and *Glycine max*), monocot angiosperms (e.g., *Oryza sativa*, *Triticum aestivum*, and *Zea mays*), gymnosperms (e.g., *Pinus lambertiana*, *Ginkgo biloba*), ferns (e.g., *Ceratopteris richardii*, *Selaginella moellendorffii*), bryophytes (e.g., *Physcomitrella patens*, *Sphagnum palustre*, and *Marchantia polymorpha*), as well as the streptophyte alga *Klebsormidium nitens* and the green alga *Volvox carteri* (Secco et al. 2012; Nezamivand-Chegini et al. 2021; Kumar et al. 2019; Xiao et al. 2021). The resulting phylogenetic tree resolved SPX domain proteins into four major groups: SPX, SPX-EXS, SPX-MFS, and SPX-RING, consistent with previous reports in model plants such as *Arabidopsis thaliana* and *Oryza sativa*. However, the detailed topology provides profound insights into the evolutionary trajectory of this gene family (Fig. 1).

Phylogenetic analysis reveals that the SPX gene family exhibits core evolutionary characteristics of polyphyletic origin and lineage-specific divergence, with its four subfamilies displaying distinct patterns of emergence and distribution. The SPX and SPX-RING subfamilies both originated in the chlorophyte *V. carteri*. Within the SPX subfamily, the *V. carteri* gene *Vocar.0005s0393* forms the basal clade, followed by the detection of homologous sequences in streptophyte algae and gradual divergence in terrestrial plant lineages. Bryophytes retain only a limited number of SPX genes, while progressive expansion is observed in ferns, gymnosperms, and angiosperms. In angiosperms, species such as *Arabidopsis thaliana* (*AtSPX1–4*), *Oryza sativa* (*OsSPX1–6*), and *Glycine max* (*GmSPX1–10*) form species-specific clades, which may be associated with the increasing complexity of Pi signaling networks. In contrast, the SPX-RING subfamily shows discontinuous distribution: although also originating from *V. carteri*, it is absent in streptophyte algae and most bryophytes, with the earliest homolog in land plants found in *P. patens* and subsequent vascular plants (Fig. 1). This pattern implies lineage-specific gene loss and subsequent independent expansion in vascular plants.

The SPX-EXS subfamily originated later, in streptophyte algae (e.g., *kfl00633_0050_v1.1* in *K. nitens*), and represents a clade with continuous presence and significant functional specialization. This subfamily has been conserved from streptophyte algae to angiosperms without lineage loss. Bryophyte homologs show high sequence conservation and are primarily involved in basic intercellular P transport in gametophytes (Wang et al. 2008). In ferns, SPX-EXS copy numbers increase and initial functional divergence occurs, likely adapting to the demands of rhizome P uptake and long-distance transport in emerging vascular tissues. In angiosperms, this subfamily undergoes pronounced lineage-specific expansion and functional diversification. In monocots, genes form species-specific clades with core members involved in root-to-shoot P transport and low-P stress responses (Secco et al. 2010). In dicots, genes further undergo functional specialization, regulating P metabolism throughout the plant life cycle—including root uptake, seed accumulation, and pollen tube nutrition. This functional divergence is closely tied to the co-evolution of the Pi-sensing SPX domain and the transport function of the EXS domain.

In striking contrast to the aforementioned algae-derived subfamilies, no homologous genes of the SPX-MFS subfamily have been detected in algal lineages, with its earliest occurrence documented in bryophytes. At this stage, the SPX-MFS subfamily contains a small number of genes, retaining only core homologous sequences with limited sequence divergence (i.e., conserved functional motifs), suggesting that its primitive function is related to the basic regulatory processes of Pi metabolism in early terrestrial plants. During fern evolution, the SPX-MFS subfamily begins to undergo initial divergence with a significant increase in gene sequence diversity, an evolutionary trait likely closely associated with the Pi acquisition requirements of ferns adapting to diverse terrestrial microhabitats (e.g., humus-rich forest floors, rocky outcrops). In angiosperms, the SPX-MFS subfamily exhibits remarkable lineage-specific expansion and functional divergence, a process that may rely on the functional synergy between the Pi-sensing function of the SPX domain and the transport function of the MFS domain. This provides a molecular mechanism for angiosperms to adapt to heterogeneous Pi nutrient environments (e.g., low-Pi stress, high-Pi stress), thereby facilitating the refinement and diversification of plant Pi uptake, transport, and homeostasis networks.

## Central roles of SPX-domain proteins in phosphate homeostasis

### The SPX-PHR module: a central signaling hub

PHOSPHATE STARVATION RESPONSE 1 (PHR1), a member of the GARP transcription factor family, harbors an MYB-like DNA-binding domain and a coiled-coil domain. It is homologous to PSR1, a key regulator of phosphorus homeostasis in the green alga *Chlamydomonas reinhardtii* (Moseley et al. 2006; Rubio et al. 2001 Wykoff 1999). PHR1 functions by specifically recognizing and binding to the PHR1-binding sequence (P1BS) *cis*-acting element (5′-GNATATNC-3′) in the promoters of target genes, thereby activating transcription of numerous Pi starvation-induced (PSI) genes. These targets include genes encoding phosphate transporters (PHTs), specific microRNAs (e.g., miR399), the Pi transport facilitator PHF1, and the non-coding RNA *IPS1*. This transcriptional network coordinates adaptive responses to Pi deficiency (Bustos et al. 2010; Liu et al. 2014; Rubio et al. 2001). PHR1 has multiple homologs in plants, including PHR1-LIKE1 (PHL1), PHL2, and PHL3, which act synergistically within the transcriptional network governing the plant PSR (Wang et al. 2023, 2024b). Notably, PHL1 exhibits functional redundancy with PHR1 in regulating a subset of PSI genes (Bustos et al. 2010). PHR1 orthologs have been functionally characterized in various plant species, such as rice OsPHR2, which plays a central role in regulating Pi starvation responses and Pi accumulation in shoots (Zhou et al. 2008), maize ZmPHR1 (Wang et al. 2013b), and soybean GmPHR1 (Lu et al. 2020). These findings show that the PHR1-centered transcriptional regulatory mechanism is highly conserved across plant lineages.

SPX-domain proteins function as central molecular hubs in the surveillance and maintenance of plant Pi homeostasis through the core SPX-PHR regulatory module. Under Pi-replete conditions, SPX proteins interact with and inhibit PHR transcription factors, primarily by sequestering them in the cytoplasm, thereby suppressing the expression of PSI genes. Under Pi deficiency, this inhibitory interaction is relieved, allowing PHRs to translocate into the nucleus, bind to the P1BS in target promoters, and activate a comprehensive transcriptional program that enhances Pi acquisition, translocation, and utilization (Puga et al. 2014; Lv et al. 2014; Dong et al. 2019; Osorio et al. 2019; Wang et al. 2014; Liu et al. 2010). This conserved “sensor-inhibitor-activator” circuit is a hallmark of Pi signaling, establishing the SPX-PHR module as the central system for Pi sensing and adaptive responses in plants.

Despite conservation of this core framework, the operational mechanisms show considerable variety across species, reflecting distinct evolutionary trajectories. In the model dicot *Arabidopsis thaliana*, AtSPX1 and AtSPX2 function as nuclear, phosphate-dependent inhibitors that directly interact with AtPHR1 and block its binding to target promoters, thereby regulating Pi homeostasis (Puga et al. 2014). AtSPX4 localizes to the cytoplasm and sequesters AtPHR1 to prevent its nuclear translocation (Osorio et al. 2019). A recent study further revealed that AtSPX1 can reciprocally bind to P1BS DNA and inositol polyphosphates with similar affinity, suggesting a more complex regulatory mechanism than previously appreciated (Whitfield et al. 2026).

In rice, different SPX members have evolved specialized regulatory strategies to control OsPHR2 activity. OsSPX1 and OsSPX2 localize to the nucleus and function as phosphate-dependent inhibitors that directly interact with OsPHR2, blocking its binding to the P1BS motif (Wang et al. 2014). OsSPX4 sequesters OsPHR2 in the cytoplasm under Pi-sufficient conditions and is rapidly degraded by two RING-type E3 ubiquitin ligases upon Pi starvation, thereby releasing OsPHR2 to activate PSR genes (Lv et al. 2014; Ruan et al. 2019). OsSPX6 employs a dual inhibitory mechanism, acting both in the nucleus and cytoplasm, and functions additively with OsSPX4 to fine-tune OsPHR2 activity (Zhong et al. 2018). OsSPX3 and OsSPX5 function redundantly through homodimerization and heterodimerization to modulate Pi homeostasis (Shi et al. 2014).

Similar SPX-PHR negative regulatory modules have been functionally characterized in other major crops, demonstrating the module’s conserved core logic amid mechanistic variation. In maize, ZmSPX1 interacts with ZmPHR1/2 to form a negative regulatory circuit (Luo et al. 2024). In cotton, GhSPX1-1 and GhSPX1-2 form specific pairs with GhPHR1A and GhPHL1A, respectively, constituting key components of a negative feedback loop in Pi signaling (Mulati et al. 2025). In barley, HvSPX4 represses the expression of Pi starvation-inducible genes, including the phosphate transporter gene *HvPHT1;6*, through direct interaction with transcription factors HvPHR1, HvPHR2, and HvPHR4 (Zhang et al. 2025). In Medicago truncatula, MtSPX1 and MtSPX3 regulate phosphate homeostasis and mycorrhizal colonization (Wang et al. 2021), and the MtSPX1/3–MtPHR2 network has recently been shown to orchestrate root nodulation-dependent nitrogen acquisition by controlling flavonoid biosynthesis (Wang et al. 2026). In soybean, GmSPX3 functions as a positive regulator in the phosphorus signaling network, contributing to Pi homeostasis (Yao et al. 2014a). In common bean, PvSPX1 is induced by Pi starvation and positively regulates downstream Pi-responsive genes under the control of PvPHR1 (Yao et al. 2014b).

Beyond protein-level regulation, Ge et al. (2025) revealed a novel post-transcriptional regulatory mechanism controlling SPX protein function in tomato. Under Pi starvation, a *cis*-natural antisense RNA (*cis*-NAT), designated *NAT*_*SPX5*_, is transcriptionally activated from the *SlSPX5* locus. This *cis*-NAT transcription induces RNA polymerase II (Pol II) pausing at the 5’ end of the overlapping *SlSPX5* sense gene. The paused Pol II undergoes increased Ser2 phosphorylation (Ser2p) on its C-terminal domain (CTD)—a modification that recruits the cleavage and polyadenylation (CPA) complex. Consequently, *SlSPX5* pre-mRNA undergoes preferential proximal polyadenylation, generating truncated, non-functional mRNA isoforms and thereby reducing the abundance of full-length, functional SlSPX5 protein. The reduction of SlSPX5 alleviates its inhibitory sequestration of the transcription factor SlPHL1 in the cytoplasm. Notably, the SlSPX5-SlPHL1 interaction is constitutive and independent of cellular Pi status. This mechanism represents a significant departure from the canonical Pi-dependent SPX-PHR protein–protein interaction observed in *Arabidopsis* and rice, revealing an additional layer of regulatory complexity in the plant Pi signaling network (Ge et al. 2025).

Collectively, evidence from both canonical and novel pathways strongly supports the role of SPX-domain proteins as central molecular sensors of cellular phosphate status. Their regulatory mechanisms exhibit remarkable diversity, ranging from modulation of protein–protein interactions and controlled protein degradation to transcriptional and post-transcriptional control. This mechanistic versatility underscores the evolutionary plasticity of the core SPX signaling module, enabling plants to fine-tune phosphate homeostasis strategies according to specific physiological and ecological demands of different species.

### SPX proteins as sensors: specific perception of inositol pyrophosphates

Inositol phosphates (InsPs) constitute an evolutionarily conserved class of eukaryotic signaling molecules, ranging from monophosphorylated inositol (InsP₁) to the fully phosphorylated inositol hexaphosphate (phytic acid, InsP₆). These molecules are generated through reversible phosphorylation at specific positions on the six-carbon inositol ring. The number and spatial configuration of phosphate groups collectively establish an information-encoding system, often described as a "chemical signal language" that conveys specific cellular signals (Shears and Wang 2019; Hatch and York 2010; Chakraborty et al. 2011; Cridland and Gillaspy 2020).

InsP₆ can be further pyrophosphorylated to produce inositol pyrophosphates (PP-InsPs). Despite their exceptionally low intracellular abundance, these high-energy signaling molecules exert pleiotropic regulatory effects on crucial eukaryotic processes, including development, metabolic homeostasis, and signal transduction. In plants, InsP₆ functions as a major Pi reserve, accumulating to high levels in seeds as phytate (Secco et al. 2017). InsP₆ is converted to InsP₇ via catalysis by inositol tetrakisphosphate 1-kinase 1 (ITPK1), and InsP₇ is subsequently pyrophosphorylated by diphosphoinositol pentakisphosphate kinase VIH1/VIH2 to generate InsP₈ (Dong et al. 2019; Zhu et al. 2019). These PP-InsPs, particularly InsP_8_, have been identified as central signaling molecules in plant Pi homeostasis regulation, despite their extremely low cellular concentrations. They therefore establish the upstream signaling framework that enables SPX domain proteins to perceive intracellular Pi status.

The SPX domain serves as a key module for sensing intracellular PP-InsPs. Structural analyses reveal that conserved basic residues within this domain form a positively charged surface pocket, exhibiting spatial and electrostatic complementarity to polyanionic PP-InsPs (e.g., InsP₆, InsP₇, and InsP₈) (Wild et al. 2016). Although this pocket can bind inorganic Pi with low millimolar affinity (Kd ≈ 5–20 mM), its high-affinity physiological ligands are PP-InsPs, which bind with nanomolar to micromolar affinity (Kd ≈ 50 nM–100 μM) (Jung et al. 2018). The crystal structure of the rice OsSPX1-PHR2 complex bound to InsP₆ confirmed this conserved mechanism, revealing that InsP₆ binds to a highly basic surface groove formed between helices α1, α2, and α4 of SPX1, with key residues including K29, K147 and K151 critical for InsP₆ recognition (Zhou et al. 2021). This specific binding induces a conformational change in the SPX domain, enhancing its interaction with PHR transcription factors and ultimately repressing their transcriptional activity under Pi-replete conditions—a ligand-induced allosteric mechanism underpinning SPX-mediated Pi homeostasis (Dong et al. 2019; Zhu et al. 2019; Ried et al. 2021).

**Fig. 2:**
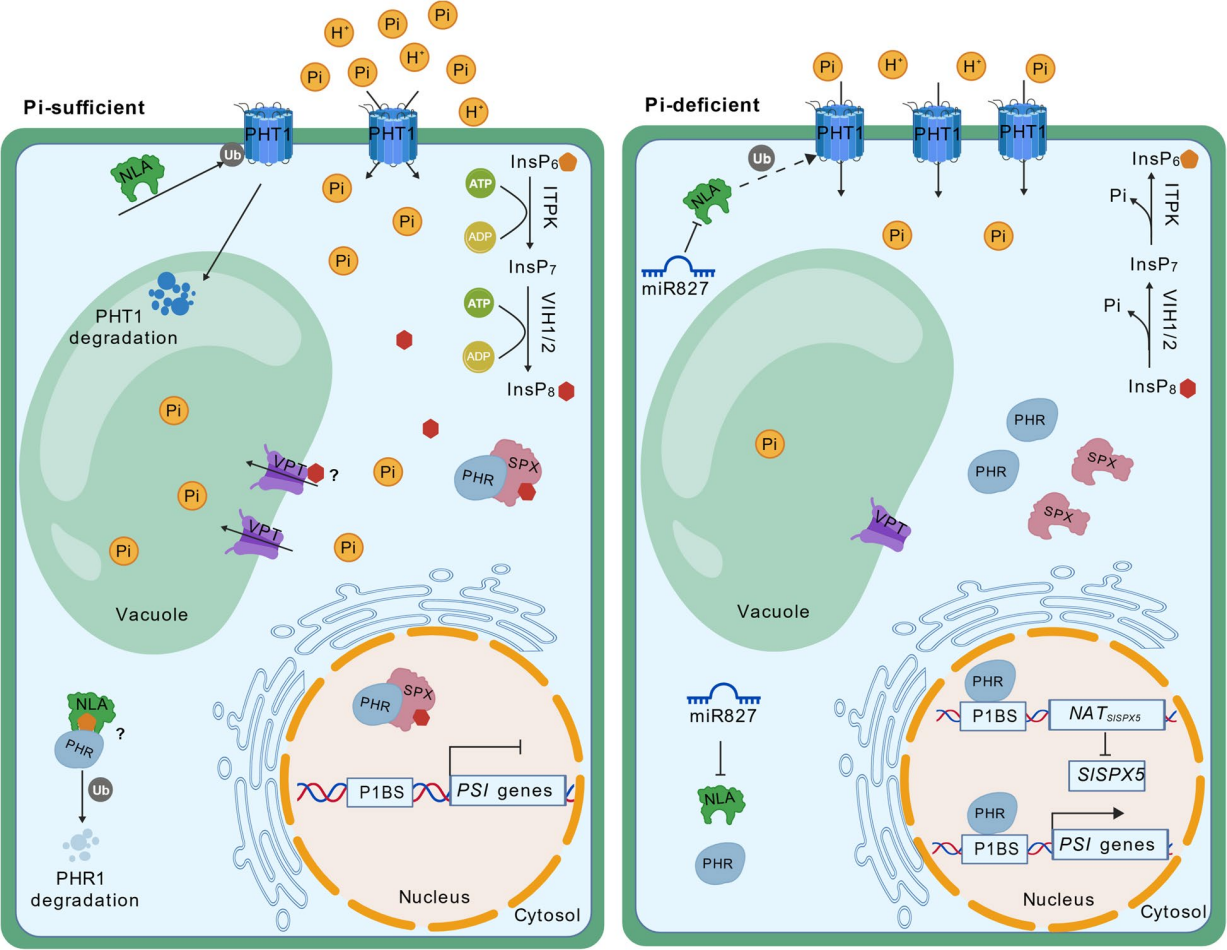
Regulatory mechanism of phosphate homeostasis by SPX domain proteins and InsP₈. Schematic model illustrating the InsP₈–SPX–PHR signaling module. Under Pi-sufficient conditions (left), elevated InsP₈ levels promote SPX–PHR complex formation, leading to repression of phosphate starvation response (PSR) genes. Under Pi-deficient conditions (right), reduced InsP₈ levels result in dissociation of the SPX–PHR complex, allowing PHR transcription factors to activate PSR gene expression. In tomato, SlPHL1 can bind to and promote the expression of *NAT*_*SlSPX5*_*,* which in turn suppresses the expression of *SlSPX5*, thereby relieving the repression on PSI genes. P1BS, PHR1-binding sequence. The templates were obtained from BioGDP (https://biogdp.com) (Jiang et al. 2025)

In the regulatory network of plant Pi homeostasis, the InsP₈–SPX–PHR module functions as a central signaling hub. Under Pi-sufficient conditions, elevated kinase activity (allosterically regulated by ATP) promotes InsP₈ synthesis. The binding of InsP₈ to SPX proteins facilitates the formation of a repressive SPX–PHR complex, primarily through interaction with the PHR coiled-coil (CC) domain, thereby suppressing transcription of PSI genes. Conversely, Pi deficiency reduces ATP levels and kinase activity, leading to decreased InsP₈ production. The consequent dissociation of the SPX–PHR complex releases PHR dimers, which then bind to P1BS motifs in the promoters of PSR genes to activate their transcription (Zhu et al. 2019; Dong et al. 2019; Ried et al. 2021) (Fig. 2).

Species-specific refinements of this core mechanism have been elucidated. The InsP₆-bound SPX1-PHR2 crystal structure from rice provided mechanistic insight into how OsSPX1 represses OsPHR2 activity. InsP₆ binding stabilizes helix α1 of OsSPX1, which sterically interferes with PHR2 dimerization. In addition, OsSPX1 directly interacts with the MYB domain of PHR2, thereby competing with DNA binding (Zhou et al. 2021; Ried et al. 2021). The essential role of InsPs sensing is underscored by mutations in the InsP₆-binding site, which disrupt OsSPX1-OsPHR2 interaction and lead to Pi accumulation and enhanced expression of PSI genes (Zhou et al. 2021). In tomato, myo-inositol phosphate synthase 2 (SlMIPS2) modulates InsP_6_ level, thereby influencing the SlSPX2–SlPHL1 interaction. This pathway integrates intracellular Pi sensing with the arbuscular mycorrhizal symbiosis, allowing coordinated adjustment of Pi homeostasis and symbiotic establishment in response to environmental Pi fluctuations (Roychowdhury et al. 2025). These advances underscore the evolutionary plasticity of the InsPs–SPX–PHR module and open new avenues for developing crops with improved PUE.

### Class II, III, and IV SPX domain proteins: coordinated regulation of phosphate homeostasis

The SPX domain protein family in plants exhibits a clear division of labor between sensing and execution. Class I proteins (SPX-only) function as the primary intracellular phosphate sensors, perceiving cellular Pi status through inositol pyrophosphate binding and transducing this signal by modulating PHR transcription factor activity (Secco et al. 2012; Liu et al. 2018). In contrast, Class II (SPX-EXS), III (SPX-MFS), and IV (SPX-RING) proteins act as effectors that directly execute Pi transport, storage, and recycling (Secco et al. 2012; Liu et al. 2018).

Many genes encoding these effector proteins are transcriptionally regulated by the PHR pathway, and the intrinsic activities of these proteins directly determine cellular and whole-plant Pi status. This Pi status, in turn, feeds back to influence the Class I sensing module, creating an integrated, multi-layered regulatory network rather than a simple linear cascade (Collins et al. 2024). Thus, Class II-IV proteins operate in concert with the Class I-based sensing hub, translating perceived Pi status into precise physiological adjustments at the cellular, tissue, and whole-plant levels.

#### Class II (SPX-EXS): long-distance phosphate transport

SPX-EXS proteins, characterized by an N-terminal SPX domain and a C-terminal transmembrane EXS domain, are key mediators of Pi translocation from roots to shoots. In *Arabidopsis thaliana*, AtPHO1 and its homolog AtPHO1;H1 facilitate Pi loading into the xylem (Hamburger et al. 2002; Stefanovic et al. 2007, 2011; Poirier et al. 1991). These proteins primarily reside in the Golgi apparatus and trans-Golgi network but can translocate to the plasma membrane (PM) under high Pi conditions to mediate efflux through an as-yet-unknown mechanism (Arpat et al. 2012; Vetal and Poirier 2023). This function is widely conserved: rice OsPHO1;2 participates in root-to-shoot Pi transport and plays a critical role in allocating Pi to developing grains, with *ospho1;2* mutants showing reduced grain yield (Secco et al. 2010; Che et al. 2020; Ma et al. 2021), while *Medicago truncatula* MtPHO1 mediates Pi transfer to symbiotic bacteroids, thereby affecting nitrogen fixation efficiency (Nguyen et al. 2021). The regulation of SPX-EXS proteins by InsPs is an evolutionarily conserved mechanism. In mammals, Pi efflux mediated by the PHO1 homolog XPR1 is regulated by intracellular InsP₈, and its dysregulation may accelerate biomineralization (Li et al. 2020). Similarly, in *Drosophila*, the Pi-sensitive orthologue PXo senses InsPs to deliver Pi to multilamellar PXo bodies. In plants, this conserved regulatory mechanism has been structurally elucidated through recent cryo-EM analysis. The structure of AtPHO1;H1 in complex with InsP₆ revealed that InsPs binding to the SPX domain promotes dimerization and activates Pi transport activity (Fang et al. 2025).

#### Class III (SPX-MFS): vacuolar phosphate storage

SPX-MFS proteins (also designated PHT5 or VPT) are tonoplast-localized transporters responsible for sequestering Pi into the vacuole. In *Arabidopsis*, AtVPT/AtPHT5 loss-of-function mutants exhibit reduced vacuolar Pi content, while overexpression lines show increased vacuolar Pi accumulation (Liu et al. 2015, 2016; Luan et al. 2019). The rice SPX-MFS family comprises three members (OsSPX-MFS1–3), with OsSPX-MFS3 serving as the predominant vacuolar Pi influx transporter (Xu et al. 2019; Guo et al. 2023b). These proteins sense cellular Pi status through their SPX domain via a sophisticated regulatory mechanism. Under Pi-sufficient conditions, InsPs binding to the SPX domain releases its autoinhibitory effect on the MFS domain. This conformational change allows SNARE proteins OsSYP21/22 to interact with the MFS domain, thereby promoting tonoplast localization and facilitating vacuolar Pi sequestration. Conversely, under Pi-deficient conditions, the SPX domain spontaneously engages in an intramolecular interaction with the MFS domain. This interaction blocks OsSYP21/22 binding, leading to retention of the transporter in the prevacuolar compartment, which reduces Pi influx and helps maintain cytosolic Pi homeostasis (Guo et al. 2023a).

#### Class IV (SPX-RING): ubiquitin-mediated regulation of transporters

SPX-RING proteins feature an N-terminal SPX domain and a C-terminal RING domain, regulating the stability of phosphate transporters (PHTs) through the ubiquitin–proteasome pathway. The PHT family encompasses numerous members that are crucial for phosphate uptake and allocation and their activity is subject to multi-layered regulation (Liu et al. 2011; Chen et al. 2011, 2015). In *Arabidopsis*, AtNLA targets PM-localized PHT1 for degradation to prevent excessive Pi uptake (Kant et al. 2011; Lin et al. 2013). Furthermore, AtNLA perceives cellular InsP₆ levels: under Pi sufficiency, elevated InsP₆ levels promote AtNLA binding to PHR1, leading to PHR1 ubiquitination and degradation, which suppresses PSR gene expression. Under Pi deficiency, decreased InsP₆ and miR827-mediated cleavage of *AtNLA* transcript release this inhibition, allowing PSR activation (Park et al. 2023). This mechanism is conserved in rice, where OsNLA1 promotes the degradation of OsPT2 and OsPT8 (Yue et al. 2017). In addition to regulating phosphate transporter stability, OsNLA1 also influences root system architecture and Pi remobilization, highlighting its multifaceted role in Pi homeostasis (Yang et al. 2017).

In summary, Class II to IV SPX proteins collectively form a precisely coordinated phosphate homeostasis execution network by mediating long-distance transport, vacuolar storage, and protein turnover, respectively. This network operates in concert with the core InsPs-SPX-PHR sensing module, enabling plants to dynamically integrate phosphate signals from the organelle to the whole-plant level and flexibly regulate Pi uptake, allocation, storage, and remobilization. A key future research direction involves elucidating the dynamic interaction patterns within this execution network and between the network and the sensing module, which will establish a theoretical foundation for the directed improvement of crop phosphorus use efficiency through synthetic biology strategies.

## Roles of SPX domain proteins in plant adaptation to biotic and abiotic stresses

### Functions in arbuscular mycorrhizal symbiosis

Most flowering plants form symbiotic associations with arbuscular mycorrhizal (AM) fungi to enhance nutrient acquisition (Abell 2017; Ezawa and Saito 2018). In this mutualistic relationship, the fungal partner explores the soil through an extensive extraradical hyphal network, acquiring phosphate via high-affinity transporters and transferring it to the plant primarily as polyphosphate (Nussaume and Kanno 2024). In exchange, plants supply fungi with photosynthetically fixed carbon, such as sugars and lipids. Emerging evidence indicates that intracellular SPX domain proteins, which function as phosphate sensors, play a pivotal role in orchestrating nutrient exchange and symbiotic establishment within this intricate network.

**Fig. 3:**
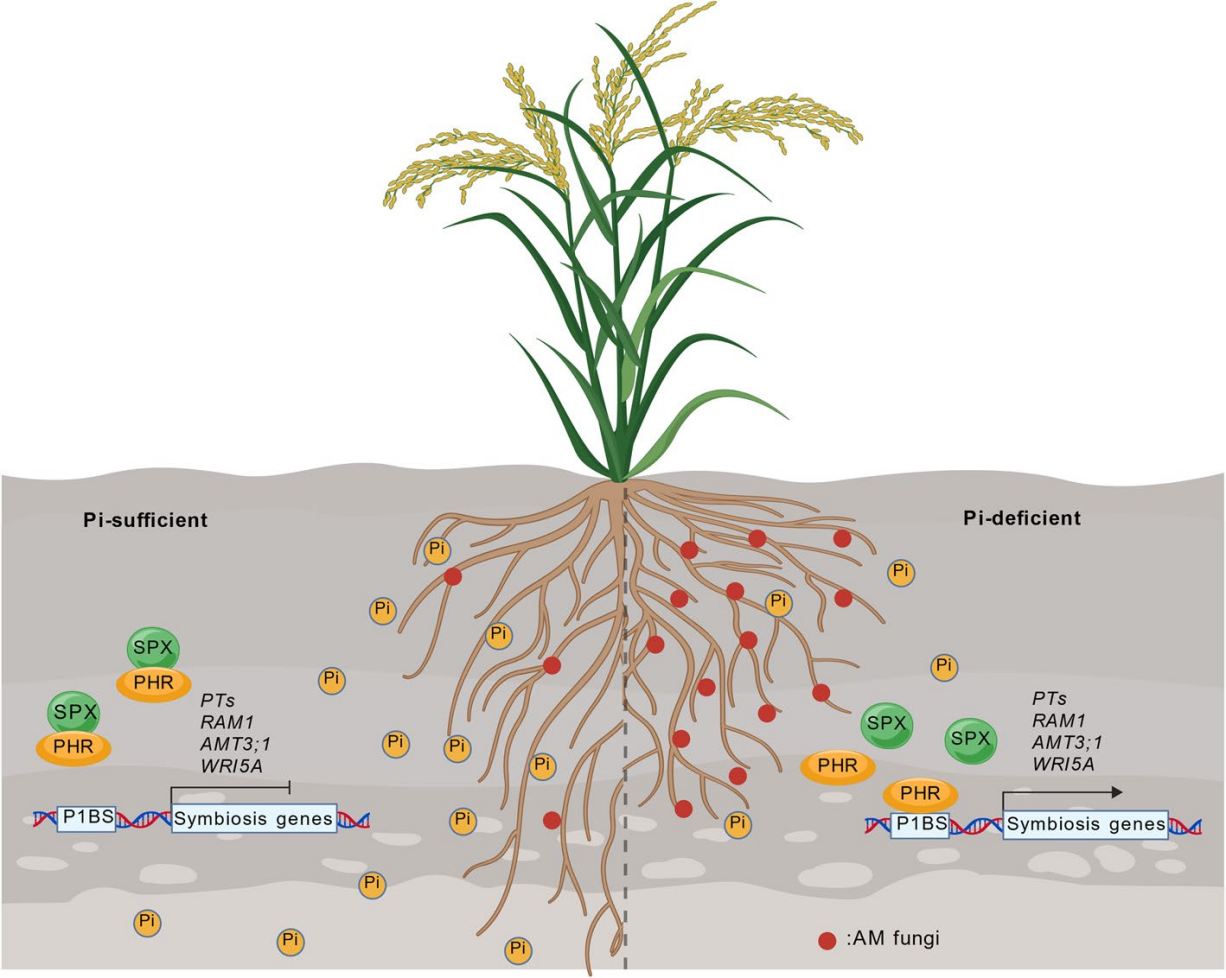
SPX proteins act as central hubs integrating phosphate signaling and arbuscular mycorrhizal symbiosis. Model depicting the dual role of SPX proteins in regulating both Pi homeostasis and AM symbiosis. Under high Pi conditions, SPX proteins repress PHR activity, leading to downregulation of symbiosis-related genes and reduced AM colonization. Under low Pi conditions, reduced SPX–PHR interaction allows activation of both PSR genes and mycorrhiza-specific phosphate transporters (e.g., PT4/PT11), thereby promoting AM colonization and Pi acquisition. The templates were obtained from BioGDP (https://biogdp.com) (Jiang et al. 2025)

SPX proteins primarily regulate AM symbiosis via interactions with PHR transcription factors, with a conserved regulatory logic across model and crop species. Under high Pi conditions, SPX proteins (e.g., SlSPX1/2 in tomato, OsSPX1/2 in rice) directly bind PHR proteins to repress their transcriptional activity. This binding leads to downregulation of symbiosis-related genes, including mycorrhiza-specific Pi transporters (SlPT4 in tomato, OsPT11 in rice), thereby suppressing AM fungal colonization. Genetic validation supports this model: SPX knockout mutants exhibit enhanced mycorrhizal colonization, whereas SPX-overexpressing lines show drastically reduced colonization (Shi et al. 2021; Singh et al. 2023; Liao et al. 2022). In *Medicago truncatula*, a legume model species, SPX proteins exhibit multifaceted, context-dependent roles that extend beyond canonical PHR repression. Under high Pi conditions, MtSPX1 and MtSPX3 repress MtPHR2 activity to maintain Pi homeostasis. Conversely, under low Pi conditions, these proteins positively regulate early symbiotic events by promoting the synthesis of strigolactones, which are essential symbiotic signals (Wang et al. 2021). Intriguingly, after symbiosis establishment, MtSPX1 and MtSPX3 localize to arbusculated cells and function as "arbuscule lifespan timers," promoting arbuscule degeneration through a partially MtPHR2-independent mechanism (Wang et al. 2024a). Taken together, these findings demonstrate that SPX proteins function not only as canonical repressors of PHR-mediated Pi signaling but also as direct regulators of the AM symbiosis program, with functional diversification across plant lineages (Fig. 3).

### Functions in nitrogen-phosphorus interactions

Plant growth and development depend on the coordinated acquisition and homeostasis of macronutrients, with nitrogen (N) and phosphorus (P) being particularly critical. The balance between these nutrients affects uptake efficiency and represents a key determinant of crop productivity (Güsewell 2004; Luo et al. 2016; Tang et al. 2025). Plants have evolved sophisticated regulatory networks to optimize N and P utilization under fluctuating nutrient conditions, and elucidating these mechanisms is essential for refining agricultural nutrient management strategies.

The nitrate signaling pathway is well-characterized in plants such as *Arabidopsis* and rice. A core component is AtNRT1.1 (also known as CHL1 or AtNPF6.3), which functions both as a dual-affinity nitrate transporter and a nitrate sensor (Ho et al. 2009). Under nitrate limitation, phosphorylation switches AtNRT1.1 to a high-affinity state, initiating nitrate signal transduction (Liu and Tsay 2003). Downstream, the transcription factor AtNLP7 (NIN-LIKE PROTEIN 7) acts as a master regulator: nitrate availability triggers calcium signals that promote the nuclear translocation of AtNLP7 via Ca^2^⁺-sensor protein kinases. In the nucleus, AtNLP7 activates the expression of transcriptional repressors such as HRS1/HHOs/NIGT1s, which subsequently repress nitrate starvation response (NSR) genes. Conversely, nitrate deficiency leads to cytoplasmic retention of AtNLP7, inhibiting the nitrate signaling pathway and thereby derepressing NSR genes (Kiba et al. 2018; Medici et al. 2019; Liu et al. 2017, 2022).

Emerging evidence indicates that SPX domain proteins are integrated into N-P crosstalk networks via multi-layered regulation by core N-signaling components, at both transcriptional and post-translational levels (Nussaume and Kanno 2024). At the transcriptional level, N-signaling-activated repressors (AtHRS1/HHOs/NIGT1s in *Arabidopsis*) directly bind promoters of *AtSPX1*, *AtSPX2*, and *AtSPX4*, repressing their expression. This relieves SPX-mediated inhibition of AtPHR1, thereby enhancing PHR1-dependent PSR (Ueda et al. 2020). In *Solanum pennellii*, *SpSPX1* and *SpSPX3* are strongly induced under N deficiency, underscoring a conserved role for SPX proteins in plant adaptation to low-N conditions (Yang et al. 2022). At the post-translational level, the rice nitrate transporter OsNRT1.1B interacts with OsSPX4 and recruits the E3 ubiquitin ligase OsNBIP1 to promote OsSPX4 ubiquitination and degradation. Since OsSPX4 modulates nucleo-cytoplasmic partitioning of both OsPHR2 (Pi signaling) and OsNLP3 (nitrate signaling), the OsNRT1.1B-OsSPX4 cascade constitutes a pivotal module integrating N and P signaling pathways (Hu et al. 2019; Zeng et al. 2025).

### Functions in low-temperature stress responses

Low-temperature stress includes both chilling (0–15 °C) and freezing (< 0 °C) stress. Freezing stress induces ice formation both intracellularly and extracellularly, leading to protoplast dehydration. Chilling stress impairs enzyme activities and disrupts cellular processes across various metabolic pathways, ultimately causing metabolic imbalance (Guo et al. 2018; Ding et al. 2019). The application of phosphorus enhances plasma membrane integrity and increases activity of antioxidant enzymes, such as superoxide dismutase (SOD), peroxidase (POD), and catalase (CAT), thereby improving chilling tolerance in rice and other species (Ihtisham et al. 2023). Therefore, P fertilization represents a common agronomic practice to enhance plant resilience against low-temperature stress. Recent studies have uncovered that SPX domain proteins mediate Pi-dependent cold tolerance, with their expression dynamically regulated by low-temperature stress.

In rice, multiple *OsSPX* genes (*OsSPX1*, *OsSPX2*, *OsSPX4* and *OsSPX6*) are upregulated under cold stress, and their promoters are enriched with cis-elements associated with abiotic stress tolerance and hormone signaling—patterns mirroring the expression patterns of known cold-tolerance genes (Huang et al. 2023). Functional characterization of OsSPX1 revealed that its overexpression significantly enhances chilling tolerance in *Arabidopsis* and tobacco, concomitant with reduced leaf total P content (Zhao et al. 2009). Conversely, OsSPX1 silencing increases plant sensitivity to chilling and oxidative stress, likely due to aberrant Pi accumulation and misregulation of oxidative stress-responsive genes (Wang et al. 2013a). In cotton, genome-wide association analysis identified *GhSPX9* as a key cold-tolerance gene: GhSPX9 silencing drastically increases cold sensitivity during germination and seedling development stages (Lin et al. 2025).

Beyond transcriptional regulation, a recent study in maize has elucidated a post-translational mechanism by which the SPX-RING E3 ligase NLA coordinates cold tolerance with phosphate homeostasis (Liao et al. 2026). Under cold stress, NLA protein is stabilized and subsequently promotes the degradation of JAZ11, a repressor of jasmonate (JA) signaling, thereby activating JA-mediated cold responses. Concurrently, NLA targets the phosphate transporter PT4 for ubiquitination and degradation in an InsPs-dependent manner, preventing excessive Pi uptake under cold conditions. This dual substrate specificity of NLA represents an elegant molecular mechanism that coordinates stress responses with nutrient homeostasis.

### Functions in integrating ABA and nitrate signals for drought response

Plants must coordinate their responses to multiple environmental stresses, yet how nutrient status and drought signals are integrated has remained poorly understood. Recent evidence has positioned OsSPX4 as a key player in this cross-talk, linking nitrogen availability to ABA-mediated stress adaptation. Abscisic acid (ABA) is the primary phytohormone mediating drought responses. A recent breakthrough revealed that the nitrate transceptor OsNRT1.1B functions as a dual receptor capable of binding both nitrate and ABA, with OsSPX4 acting as a critical node in this signaling pathway (Ma et al. 2025). Under low-nitrate conditions, ABA binding to OsNRT1.1B promotes the formation of the OsNRT1.1B-OsSPX4 complex at the plasma membrane. This interaction triggers the release of OsNLP4, a NIN-LIKE PROTEIN transcription factor that is sequestered by OsSPX4 under Pi-sufficient conditions, allowing OsNLP4 to translocate to the nucleus and activates ABA-responsive genes. This cascade thus integrates three signals — nitrate availability, phosphate status, and ABA — through the sequential action of OsNRT1.1B and OsSPX4 (Ma et al. 2025).

Genetic evidence supports this model: *osnrt1.1b* mutants exhibit impaired ABA responses under low-nitrate conditions, while OsNRT1.1B overexpression enhances drought resistance. The ABA-binding site is conserved in OsNRT1.1 homologs across higher plants, and ABA promotes OsNRT1.1-SPX interactions in Arabidopsis, maize, and wheat, indicating that this mechanism may be broadly conserved in flowering plants (Ma et al. 2025). Within this OsNRT1.1B-OsSPX4-OsNLP4 cascade, OsSPX4 plays a pivotal role by sequestering NLP4 under nitrogen-replete conditions and releasing it upon ABA-OsNRT1.1B interaction under nitrogen limitation. OsSPX4 thus functions as a central integrator that translates nitrogen status at the plasma membrane into appropriate ABA-mediated transcriptional outputs in the nucleus, exemplifying how SPX proteins coordinate nutrient sensing with abiotic stress adaptation (Collins et al. 2024; Ma et al. 2025).

### Functions in mediating growth-immunity trade-off in response to phosphate availability

Plants must constantly balance growth and defense, a trade-off that is profoundly influenced by nutrient availability. Brassinosteroids (BRs) are well-characterized growth-promoting hormones that also contribute to biotic stress responses (Lozano-Durán and Zipfel 2015; Nolan et al. 2020). In rice, the BR signaling transcription factor BRASSINAZOLE-RESISTANT 1 (OsBZR1) directly regulates the biosynthesis of sakuranetin, a phytoalexin that confers resistance against the rice blast fungus *Magnaporthe oryzae*, thereby establishing a direct molecular link between BR signaling and basal immunity (He et al. 2024).

Recent evidence has identified OsSPX1 and OsSPX2 as key regulators that coordinate growth and immunity in response to fluctuating phosphate availability. OsSPX1/2 physically interact with OsBZR1 under both phosphate-sufficient and -deficient conditions, repressing the expression of BR-responsive genes (He et al. 2024). Under phosphate-sufficient conditions, OsSPX1/2 is predominantly sequestered by OsPHR2, the master transcriptional activator of phosphate starvation responses (Wang et al. 2014). This sequestration allows OsBZR1 to remain largely active, promoting growth while maintaining basal resistance through sakuranetin accumulation. Under phosphate starvation, however, OsSPX1/2 is released from OsPHR2 and becomes available to interact with and inhibit OsBZR1, leading to reduced growth and diminished sakuranetin accumulation.

The OsBZR1-OsSPX1/2 module thus fine-tunes the growth-immunity trade-off in a phosphate-dependent manner. Genetic evidence supports this model: plants with altered OsSPX1/2 expression exhibit corresponding changes in growth rates and disease resistance under varying phosphate conditions (He et al. 2024). These findings reveal a novel function for SPX proteins in coordinating nutrient status with immune responses, adding another dimension to their role as central integrators of plant physiology.

Taken together, the studies discussed in this section establish SPX domain proteins as central hubs in plant stress biology, functioning far beyond their canonical role as intracellular phosphate sensors. By translating cellular Pi status into precise regulatory outputs, they modulate symbiotic partnerships, coordinate nitrogen–phosphorus balance, mediate cold tolerance, integrate drought and ABA signaling, and fine-tune the growth–immunity trade-off. This multifaceted functionality positions SPX proteins as master regulators that integrate nutrient status with diverse environmental signals to optimize plant fitness.

## Discussion

### Integrative insights into the SPX-Centered Regulatory Network for Pi homeostasis​

Accumulating evidence from structural, molecular, and evolutionary studies has firmly established SPX domain proteins as the central hub of plant Pi signaling and homeostasis. This review synthesizes a unified model in which SPX proteins orchestrate Pi acquisition, transport, storage, and utilization through a multi-layered regulatory network. At the core of this network lies the SPX-PHR module, whose function is conserved across all major land plant lineages, from bryophytes to angiosperms, underscoring its evolutionary importance as a fundamental Pi-sensing mechanism. However, the mechanistic diversity of this module, such as Pi-dependent protein–protein interactions in *Arabidopsis* (Wild et al. 2016), ubiquitin-mediated degradation of SPX proteins in rice (Collins et al. 2024; Ruan et al. 2019), and *cis*-NAT-regulated post-transcriptional control in tomato (Ge et al. 2025), reflects adaptive evolution to diverse ecological niches and physiological demands.

A key discovery in recent years is the identification of inositol pyrophosphates (PP-InsPs) as the upstream signaling molecules that link cellular Pi status to SPX-PHR activity. The high-affinity binding of InsP_8_ to the positively charged pocket of the SPX domain provides a molecular explanation for how plants precisely sense Pi fluctuations (Dong et al. 2019; Jung et al. 2018). Importantly, the dual regulation of PHR by SPX proteins, which involves both inhibiting its oligomerization and blocking its DNA binding, ensures robust repression of PSR genes under Pi-replete conditions, thereby preventing metabolic waste and potential Pi toxicity (Ried et al. 2021; Zhou et al. 2021). Beyond the core sensing mechanism, the functional specialization of SPX subfamilies (SPX-EXS for long-distance transport, SPX-MFS for vacuolar storage, and SPX-RING for ubiquitin-mediated degradation) reveals a division of labor that allows for spatiotemporal control of Pi homeostasis.

### SPX proteins as emerging nodes in multi-signal integration​

Beyond their canonical role in Pi signaling, SPX proteins have emerged as critical integrators of nutrient crosstalk, biotic interactions, and abiotic stress responses, expanding their significance in plant biology. The N-Pi balance regulated by SPX proteins exemplifies this integrative function. In *Arabidopsis*, NIGT1/HRS1 transcription factors repress SPX expression to enhance PHR activity under N limitation (Ueda et al. 2020), while in rice, OsNRT1.1B-mediated degradation of OsSPX4 coordinates N and Pi signaling (Hu et al. 2019). This crosstalk ensures optimal allocation of resources when either nutrient is limiting, highlighting SPX proteins as key mediators of plant nutrient use efficiency (NUE). Similarly, the role of SPX proteins in arbuscular mycorrhizal (AM) symbiosis demonstrates their ability to balance Pi acquisition via roots and symbiotic pathways. This is evidenced by their functions ranging from repressing fungal colonization under high Pi conditions (Shi et al. 2021) to regulating arbuscule lifespan in Medicago (Wang et al. 2024a). Such multifaceted regulation suggests that SPX proteins act as "signal integrators" that translate environmental cues (e.g., nutrient availability, symbiont presence) into adaptive responses, a concept that warrants further exploration in the context of other nutrient signaling pathways (e.g., sulfur, potassium).

The involvement of SPX proteins in abiotic stress tolerance, particularly cold stress, adds another layer of complexity to their functional repertoire. Overexpression of OsSPX1 enhances chilling tolerance (Zhao et al. 2009), while GhSPX9 is required for cold adaptation in cotton (Lin et al. 2025). The underlying mechanisms likely involve the integration of Pi homeostasis with redox balance and stress-responsive gene regulation, as Pi deficiency exacerbates oxidative stress (Wang et al. 2011). However, the precise link between SPX-mediated Pi sensing and stress signaling remains unclear. For example, it is not known whether SPX proteins directly interact with stress-responsive transcription factors or modulate InsP_8_ levels to influence stress signaling cascades. Addressing these questions will provide a more comprehensive understanding of how plants coordinate nutrient homeostasis and stress adaptation.

### Current challenges and unresolved questions​

Despite significant progress, several critical gaps remain in our understanding of SPX domain proteins. First, the structural basis for the remarkable functional diversity among SPX subfamilies, despite their shared conserved SPX domain, remains unclear and requires elucidation. Future investigations should employ structural biology approaches, such as X-ray crystallography and cryo-electron microscopy, to determine the three-dimensional structures of distinct SPX members. This information will be crucial for elucidating the specific mechanisms governing their ligand recognition and protein–protein interactions. Second, the role of SPX proteins in crop species is understudied relative to model plants. While orthologs of SPX genes have been identified in maize (Luo et al. 2024), wheat (Qian et al. 2025), and soybean (Lu et al. 2020; Nezamivand-Chegini et al. 2021), their functional characterization remains limited. Given the importance of Pi use efficiency (PUE) in sustainable agriculture, understanding the molecular mechanisms of SPX proteins in crops is essential for translational research applications. Third, the post-translational modifications (PTMs) of SPX proteins remain largely uncharacterized. Phosphorylation, ubiquitination, or sumoylation could modulate their stability, subcellular localization, or interaction with partners, but only a few studies have addressed this important regulatory layer. Finally, the integration of SPX signaling with other phytohormone pathways (e.g., auxin, cytokinin) that regulate root development and Pi acquisition is poorly understood. Unraveling these crosstalk mechanisms will provide a more holistic view of plant Pi homeostasis.​

### Translational perspectives for improving crop PUE​

The mechanistic understanding of SPX-mediated phosphate signaling opens promising pathways for improving phosphorus use efficiency (PUE) in crops, which is an essential objective for sustainable agriculture. Based on these discoveries, several targeted strategies can be developed to enhance crop performance under Pi-limiting conditions. One approach involves modulating SPX subfamily expression. For example, overexpressing vacuolar storage facilitators like OsSPX-MFS3 or silencing negative regulators like the SPX-RING protein NLA, can optimize Pi sequestration and transporter stability, respectively. Furthermore, engineering nutrient crosstalk by targeting key integration modules (e.g., the OsNRT1.1B-OsSPX4 module in rice) could optimize the nitrogen-phosphorus balance, reducing fertilizer dependency. Additionally, repressing specific SPX genes (e.g., SlSPX1/2 in tomato) may enhance arbuscular mycorrhizal colonization for improved Pi uptake in nutrient-poor soils. Finally, stacking SPX-mediated PUE traits with other stress-adaptation roles of SPX proteins could develop crops resilient to multiple abiotic constraints. However, translating these strategies faces challenges, including potential pleiotropic effects from constitutive PSR activation which may require inducible or tissue-specific approaches. Additionally, functional redundancy within SPX families necessitates multiplexed genome editing approaches, and field validation under diverse agronomic conditions remains imperative for practical implementation. Addressing these challenges will be crucial for realizing the full potential of SPX-based strategies in crop improvement programs.

## Supplementary Information

Supplementary Material 1.

## Data Availability

Not applicable.

## Abbreviations

AM: Arbuscular Mycorrhizal
HRS1: Hypersensitive Response Suppressor 1
IPS1: Induced by Phosphate Starvation 1
ITPK1: Inositol tetrakisphosphate 1-kinase 1
InsP_6_: Inositol hexakisphosphate
MFS: Major Facilitator Superfamily
NIGT1: Nitrate-Inducible GARP-type Transcriptional repressor 1
NLA: Nitrogen Limitation Adaptation
NLP: NIN-like protein
NUE: Nutrient Use Efficiency
OsSPX: Oryza sativa SPX
P1BS: PHR1-binding sequence
PHO1: PHOSPHATE1
PHO81: Phosphatase 81
PHR1: Phosphate Starvation Response 1
PHR2: Phosphate Starvation Response 2
PHT1: Phosphate transporter 1
PM: Plasma Membrane
POD: Peroxidase
PSI: Phosphate Starvation Inducible
PSR: Phosphate Starvation Response
PUE: Phosphorus Use Efficiency
Pi: Inorganic phosphate
RSA: Root System Architecture
SOD: Superoxide dismutase
SPX: SYG1/Pho81/XPR1
SPX1: SPX domain-containing protein 1
SPX2: SPX domain-containing protein 2
SPX3: SPX domain-containing protein 3
SPX4: SPX domain-containing protein 4
SPX5: SPX domain-containing protein 5
SPX6: SPX domain-containing protein 6
SYG1: Suppressor of Yeast gpa1
XPR1: Xenotropic and Polytropic Retrovirus receptor 1
